# Psychometric Properties of the German Version of the Self-Regulation of Eating Behavior Questionnaire

**DOI:** 10.3389/fpsyg.2021.649867

**Published:** 2021-03-12

**Authors:** Ileana Schmalbach, Bjarne Schmalbach, Markus Zenger, Katja Petrowski, Manfred Beutel, Anja Hilbert, Elmar Brähler

**Affiliations:** ^1^Department of Medical Psychology and Medical Sociology, University Medical Center of the Johannes Gutenberg University Mainz, Mainz, Germany; ^2^Technische Universität Dresden, Carl Gustav Carus Faculty of Medicine, Division of Psychological and Social Medicine and Developmental Neurosciences, Research Group Applied Medical Psychology and Medical Sociology, Dresden, Germany; ^3^Applied Human Studies, University of Applied Sciences Magdeburg-Stendal, Stendal, Germany; ^4^Integrated Research and Treatment Center Adiposity Diseases, Behavioral Medicine Research Unit, Department of Psychosomatic Medicine and Psychotherapy, University of Leipzig Medical Center, Leipzig, Germany; ^5^Universitätsklinikum Carl Gustav Carus an der Technischen Universität Dresden, Abteilung für Innere Medizin III, Dresden, Germany; ^6^Department for Psychosomatic Medicine and Psychotherapy, University Medical Center of Johannes Gutenberg University of Mainz, Mainz, Germany

**Keywords:** self-regulation, eating behavior, health, short scale, psychometric properties, weight management

## Abstract

**Background:** The Self-Regulation of Eating Behavior Questionnaire (SREBQ) is an economical way of assessing an individual's self-regulatory abilities regarding eating behavior. Such scales are needed in the German population; therefore, the purpose of the present study was the translation and validation of a German version of the SREBQ.

**Method:** First, we conducted a pilot study (Study 1; *N* = 371) after the translation procedure. Second, we assessed the final scale in a representative sample of the German population (Sample 2; *N* = 2,483) and its underlying factor structure. Further, we tested for measurement invariance and evaluated the SREBQ's associations with related scales to explore convergent and discriminant validity. Finally, we considered differences in SREBQ based on sociodemographic variables and provided derived reference scores (norm values).

**Results:** Factor analysis revealed deficiencies in the original model. Thus, we shortened the scale based on statistical considerations and the adapted version showed improved fit in Confirmatory Factor Analysis and reliability. We also found evidence for partial strict invariance, which means the measure is equivalent for the tested groups of age and gender. Item and scale psychometric properties of the shortened version were satisfactory. In terms of diagnostic validity, it was shown that individuals with higher body mass index (kg/m^2^) have worse self-regulation of eating behavior than those with lower.

**Conclusion:** In sum, the SREBQ evidenced good validity and reliability and is suitable for application in medical, psychological, and nutritional research.

## Introduction

Obesity [body mass index (BMI) ≥30 kg/m^2^; Deutsche Adipositas Gesellschaft/German Obesity Society, 2014] is a serious health concern, that not only affects patient's health at an individual level (e.g., cardiovascular and metabolic diseases; Lewis et al., 2009; Swinburn et al., 2011), but it also has consequence at a societal dimension (e.g., unemployment; economic burden for health care; Lehnert et al., 2015; Effertz et al., 2016; De Lorenzo et al., 2019). Psychologically, it is associated with depression and eating disorders [e.g., binge-eating disorder (BED); Hilbert, 2021]. In Germany, the proportion of citizens with obesity has been growing since the past three decades peaking at about 24% of the population (Mensink et al., 2013). Effertz et al. (2016) reported that obesity caused by unhealthy eating habits is, after smoking, the second riskiest “lifestyle factor” resulting in poor quality of life (Deutsche Adipositas Gesellschaft/German Obesity Society, 2014; Effertz et al., 2016). Specifically, self-regulation of eating behavior has been directly related to positive physical and mental health outcomes, as well as to overall life satisfaction (Torres and Nowson, 2007; Grant et al., 2009; Gupta and Verma, 2019; Sharbafshaaer, 2019). On the other hand, low self-regulation is associated with high BMI and obesity (Balani et al., 2019; Ruzanska and Warschburger, 2019).

Individuals can positively affect their own health by enhancing self-regulated eating (Reed et al., 2016), however, this can be challenging. Therefore, the ability to effectively regulate one's eating behavior is an important goal for many health-related interventions (Gardner et al., 2012; Llewellyn and Wardle, 2015). Consequently, standardized scales for the evaluation of such interventions are key. For this purpose, Kliemann et al. (2016) developed the Self-Regulation of Eating Behavior Questionnaire (SREBQ), which assess the extent to which individuals are able to effectively self-regulate their eating behavior and measure change in response to self-regulation interventions. The aim of the present study is to develop a German version of the SREBQ and assess its psychometric properties.

### Self-Regulation of Eating Behavior

In general, behavioral self-regulation is defined as intentional control of attention, thoughts, emotions, behaviors, and environment (Carver and Scheier, 2012, 2017; Baumeister et al., 2018; Usher and Schunk, 2018) that can be improved with practice (Hofmann et al., 2012). From this perspective, individuals engage in situation selection, modification, attentional deployment, cognitive change, and response modulation of various kinds (Gross, 2014). For example, someone who is trying to lose weight might choose to refrain from situations involving enticing, calorically dense foods. Hence, by simply avoiding situations that could be detrimental to their goal (i.e., losing weight), they enhance the probability for a successful goal achievement. Other strategies related to eating behavior include self-monitoring, planning, and impulse control—among others (Kreausukon et al., 2012; Hankonen et al., 2013; Dombrowski et al., 2014). Accordingly, it has been suggested that the ability to choose and competently apply strategies of self-regulation may explain the gap between intention and behavior (Allan et al., 2011; Gellert et al., 2011). For instance, individuals lacking self-regulation in the sense of reduced impulse control, could be at higher risk of weight gain (Campos-Uscanga et al., 2017; Johnson and Annesi, 2018). In patients with obesity, studies revealed deficits in broad executive functions, which are strongly correlated with successful self-regulation (Dohle et al., 2018; Yang et al., 2018). From this perspective, affected patients may find it challenging to ingest appropriate amounts of food (according to their caloric needs), exhibiting abnormal eating habits. Similarly, in individuals with binge-eating disorder (BED) tend to overeat, while those with anorexia nervosa (AN) are prone to extremely reduce their food intake (Hagger et al., 2009; Leehr et al., 2015; Dingemans et al., 2017; Kenny et al., 2017, 2019). In this light, the ability to effectively regulate one's eating behavior is a major goal not only for patients with an ED, but also for individuals in dietary and weight-loss interventions (e.g., obesity).

Indeed, there are scales assessing self-regulation, however, regulatory aspects of eating behavior are not considered (Carey et al., 2004; Tangney et al., 2004; Schroder et al., 2013), relate to children and adolescents (Moilanen, 2007; De Vet et al., 2014; Monnery-Patris et al., 2019), are gender-specific (Pelletier et al., 2004) or lack invariance across genders (Hamilton et al., 2018). Therefore, there is a need of valid measures that measure self-regulation of eating behavior for a broader population. Hence, the main goal of the SREBQ is to evaluate the self-regulatory skills in terms of regulating eating behavior, considering the capabilities that are necessary to healthfully regulate eating (Kliemann et al., 2016).

### Purpose of the Study

The purpose of the present study was to translate the SREBQ and validate it in a German population sample. To this end, we analyzed item and scale psychometric properties, and conducted factor analysis and tests of measurement invariance. Furthermore, - to explore convergent and discriminant validity—we computed correlations between the SREBQ and several established measures. We expected better self-regulation of eating behavior to be associated with lower eating disorder psychopathology, lower overall psychological distress, lower symptoms of depression and anxiety, higher satisfaction with life, better subjective health status, and a better nutritional and weight management lifestyle. In terms of diagnostic validity, we hypothesized that individuals with a higher BMI (kg/m^2^) would exhibit lower levels of self-regulation (Cameron and Leventhal, 2003; Hagger et al., 2009; Berking and Wupperman, 2012; De Castella et al., 2013; Leventhal et al., 2016; Campos-Uscanga et al., 2017; Johnson and Annesi, 2018; Annesi, 2019).

## Method

The translation process (English items into German) was based on the WHO protocol of translation and adaption of instruments (e.g., forward and back translation, pre-testing and final version; World Health Organization, 2010). In order to improve the quality of the analyses, two samples were collected. In Sample 1, the quality of the psychometric properties of the newly translated scale were piloted (Study 1). In a validation study (Study 2) the final scale was tested in a larger and representative sample (Sample 2). See Rresults section.

### Participants and Procedure

In general, mentally and physically healthy participants of a minimum age of 14 years were included. Participants without knowledge of the German language were excluded.

#### Sample 1

We recruited a sample of *n* = 462 participants using the online survey tool SoSciSurvey (Leiner, 2014). After giving their informed consent, the participants submitted their sociodemographic information and answered several questionnaires about their mental and physical health. The average study duration was 754 seconds (s) (*SD* = 481 s). The ethics committee of the University of Applied Sciences Magdeburg-Stendal approved of the procedure (AZ-4973-75). We conducted the study in accordance with the common ethical guidelines of the Declaration of Helsinki. Ninety (19.5%) participants dropped out from the survey before or after giving their sociodemographic information, leaving *n* = 371 participants, who answered the SREBQ. Participants were on average *M* = 27.02 years old (*SD* = 8.51; *Min* = 16; *Max* = 66). Further sample characteristics are reported in Table 1. The German items are displayed in Table 2.

**Table 1:** Sociodemographic characteristics of both samples.

	**Sample 1 (*N* = 371)**			**Sample 2 (*N* = 2,483)**		
	***n***	**%**	**SREBQ^a^**	***n***	**%**	**SREBQ^a^**
**Gender/sex**						
Female	314	84.6	3.29 (0.70)	1,250	50.3	3.30 (0.77)
Male	53	14.3	3.56 (0.79)	1,233	49.7	3.16 (0.75)
Other	4	1.1	3.38 (0.60)	–	–	
**Age groups (years)**						
14–29	271	73.0	3.32 (0.71)	401	16.1	3.24 (0.80)
30–39	65	17.5	3.22 (0.72)	379	15.3	3.23 (0.77)
40–49	25	6.7	3.59 (0.77)	416	16.8	3.19 (0.78)
50–59	8	2.2	3.59 (0.55)	528	21.3	3.20 (0.74)
60–69	2	0.5	3.00 (1.41)	411	16.6	3.21 (0.79)
≥70	–	–	–	348	14.0	3.35 (0.69)
**Education**						
>10 years	–	–	–	750	30.2	3.22 (0.76)
10 years	–	–	–	1,104	44.5	3.18 (0.77)
<10 years	331	89.2	3.33 (0.72)	590	23.8	3.33 (0.74)
Other	40	10.8	3.33 (0.78)	39	1.6	3.45 (0.76)
**Family**						
Married	40	10.8	3.50 (0.66)	1,101	44.3	3.19 (0.75)
Separated	7	1.9	3.21 (1.04)	55	2.2	3.37 (0.69)
Single	306	82.5	3.30 (0.75)	726	29.2	3.22 (0.78)
Divorced	6	1.6	3.75 (0.39)	373	15.0	3.28 (0.76)
Widowed	–	–	–	223	9.0	3.34 (0.77)
Other	12	3.2	3.54 (0.68)	5	0.2	3.55 (0.62)
**Employment**						
Working full- or part-time	206	55.5	3.38 (0.71)	1,516	61.0	3.22 (0.75)
Not working	10	2.7	3.30 (0.52)	189	7.6	3.15 (0.83)
Retired	1	0.3	2.00 (–)	622	25.1	3.28 (0.71)
Student/apprentice	154	41.5	3.26 (0.74)	156	6.3	3.24 (0.84)
**Income**						
<500 €	137	36.9	3.19 (0.72)	214	8.6	3.29 (0.76)
500–999 €	90	24.3	3.38 (0.69)	431	17.4	3.13 (0.82)
1,000–1,999 €	79	21.3	3.34 (0.77)	1,225	49.3	3.22 (0.74)
2,000–3,500 €	42	11.3	3.49 (0.66)	537	21.6	3.29 (0.75)
>3,500 €	11	3.0	3.57 (0.55)	76	3.1	3.38 (0.77)
Refused to answer	12	3.2	3.58 (0.76)	–	–	–
**Weight status**						
<25 kg/m^2^	–	–	–	1,097	44.2	3.43 (0.74)
25–30 kg/m^2^	–	–	–	1,056	42.5	3.14 (0.73)
>30 kg/m^2^	–	–	–	330	13.3	2.85 (0.74)

a *Means (Standard Deviations) of the SREBQ mean score for each sociodemographic group*.

**Table 2:** SREBQ item and scale characteristics (Sample 1).

	***M***	***SD***	**γ_1_**	**γ_2_**	**λ_EFA_**	***r*_it_**	***r*_itb_**
Item 1—“Ich kann verlockendem Essen gut widerstehen”	2.72	0.97	0.122	−0.562	0.402	0.368	0.716
Item 2—“Ich werfe meine guten Ernährungsvorsätze zu leicht über Bord” ^r^	3.20	1.00	−0.006	−0.793	0.914	0.618	0.492
Item 3—“Ich lasse mich leicht von meinen Ernährungsvorsätzen ablenken” ^r^	3.26	1.04	0.000	−0.847	0.766	0.542	0.553
Item 4—“Es fällt mir abends schwer, mich daran zu erinnern, was ich im Laufe des Tages gegessen habe” ^r^	4.13	0.98	−0.971	0.138	0.398	0.365	0.721
Item 5—“Wenn ich nicht so esse, wie ich es mir eigentlich vorgenommen habe, ändere ich etwas”	2.88	1.03	−0.201	−0.721	0.226	0.237	
Original scale	3.24	0.66	−0.272	−0.013			
Adapted scale	3.33	0.72	−0.340	−0.086			

*γ_1_ = skewness; γ_2_ = excessive kurtosis; r_it_ = corrected item-total-correlation; r_itb_ = corrected item-total-correlation for the shortened scale (without Item 5); ^r^ = Item is reverse-coded*.

#### Sample 2

This sample was recruited by the demographic consulting company USUMA, Berlin, by using a multistage sampling approach, aiming for a sample that is representative of the German general population in terms of participant age and sex. First, households were selected using a random-route procedure. Within each household, the interviewee was selected based on the Kish selection grid. A trained professional then conducted the interviews with the consenting participants. Custodian consent from parents or legal guardians was granted for underaged participants (oral consent in accordance with the German law). The ethics committee of the University of Leipzig approved the procedure (002/20—ek). The resulting sample consisted of *n* = 2,503 individuals. After excluding respondents with missing values, we retained a sample of *n* = 2,483. Participants were on average *M* = 49.52 years old (*SD* = 17.47; *Min* = 14; *Max* = 95) and we found a close to even sex distribution (see Table 1). In this regard, Sample 2 was representative for the German population regarding age and sex (Federal Statistical Office of Germany, 2019). The average BMI (kg/m^2^) was *M* = 26.12 (*SD* = 4.74; *Min* = 15.94; *Max* = 66.57).

### Instruments

*Self-Regulation of Eating Behavior Questionnaire* (SREBQ; Kliemann et al., 2016). This scale assesses an individual's self-regulatory capacity in terms of eating behavior, using five items (e.g., “I'm good at resisting tempting food”). Participants answered these items on a 5-point scale from “0 = never” to “4 = always”. The scale score is obtained by calculating the sum score of the items. The psychometric properties of the SREBQ are satisfactory (Cronbach's α = 0.75; Kliemann et al., 2016). The reliability in the present study was ω = 0.919.

*Eating Disorder Examination-Questionnaire 8* (EDE-Q8; Kliem et al., 2016). The EDE-Q8 measures eating disorder psychopathology using eight items on a 7-point scale (“0” – “6”), and higher mean scores reflect greater severity or frequency. The authors reported adequate internal consistency (α = 0.93). In the present study, the reliability was ω = 0.872.

The *Symptom Checklist-K-9* (Klaghofer and Brähler, 2001; Prinz et al., 2013; SCL-9-K) captures general psychological distress using nine items on a 5-point scale “1 = not at all” to “4 = extremely.” Higher mean scores demonstrate increased symptom severity. Its psychometric properties including internal consistency are satisfactory (α = 0.84; Prinz et al., 2013). The reliability in the present study was ω = 0.768.

*Patient Health Questionnaire* (Kroenke et al., 2009; Löwe et al., 2010; PHQ-4). This tool screens for symptoms of depression and anxiety using two items each on a 4-point scale (“1 = not at all” to “4 = nearly every day”). Higher sum scores demonstrate increased symptom of depression. The psychometric properties of the PHQ-4 and Cronbach's Alpha are satisfactory (α = 0.81–82; Löwe et al., 2010). In the present study, the reliability was ω = 0.821.

The *Satisfaction-with-Life Scale* (SWLS; Glaesmer et al., 2011) uses five items on a 7-point scale (“1 = strongly agree” to “7 = strongly disagree”) to measure overall life satisfaction. We inverted the score, so that higher sum scores indicate higher life satisfaction. Glaesmer and colleagues reported adequate psychometric properties and internal consistency (α = 0.92). The reliability in the present study was ω = 0.892.

*Health Visual Analog Scale* (Health VAS; Brooks et al., 2003). Participants indicate their subjective health status on the Health VAS, ranging from 0 “worst imaginable health status” to 100 “best imaginable health status.”

*Six Factor Weight Management Questionnaire* (6FQ; Kushner et al., 2016). This scale consists of 27-items that identify six unhealthy affective, cognitive, and behavioral lifestyle factors related to nutrition and weight management (Convenient Diner, Fast Pacer, Easily Enticed Eater, Exercise Struggler, Self-Critic, All-or-Nothing Doer). Participants answer on a 4-point scale (“1 = don't agree at all” to “4 = strongly agree”). Higher sum scores indicate association with the weight management style. We translated the questionnaire using the same method as we did for the SREBQ. The psychometric properties are satisfactory. The reliability in the present study was between ω =0.731 and.861 for the six subscales.

### Statistical Analyses

We conducted the statistical analysis using R and the packages *EFAutilities, ezCutoffs, lavaan, paran*, and *semTools* (Rosseel, 2012; SemTools Contributors, 2016; Dinno, 2018; Schmalbach et al., 2019; Zhang et al., 2020). Our hypotheses and data-analytical plan were specified prior data collection. For the exploratory factor analysis (EFA), we first ran parallel analysis (PA; Horn, 1965) to establish the number of components in the data by comparing the empirical eigenvalues to those of randomly generated data sets with the same general properties. Then, we utilized EFA to check the loadings of the indicators. For the CFA (Confirmatory Factor Analysis), we used the robust maximum likelihood estimation method with Yuan-Bentler's scaled χ^2^ (Yuan and Bentler, 2000). To evaluate goodness-of-fit, we utilized popular fit indices with commonly recommended cut-off criteria for good fit (Hu and Bentler, 1998, 1999; Schermelleh-Engel et al., 2003): The χ^2^-test which should ideally not be significant; the Comparative Fit Index (CFI) and the Tucker-Lewis Index (TLI), which should both be larger than 0.95, the Root Mean Square Error of Approximation (RMSEA) and its 90% confidence interval and the Standardized Root Means Square Residual (SRMR) which should both be smaller than 0.08. To supplement these model evaluations, we employed the simulation-based approach introduced by Schmalbach et al. (2019): By simulating and fitting data sets based on the parameter tables of the empirical model at hand and then finding the 95th percentile of the resulting fit indices, one can obtain empirically justified fit index cutoffs. Now, if the empirical fit index is within the bounds set out by the simulated cutoff, this is strong evidence for good model fit.

Furthermore, we tested measurement invariance across age, gender, and BMI (kg/m^2^) status using the procedure described by Milfont and Fischer (2010): We compared increasingly constrained models in a stepwise fashion to establish increasingly strict levels of invariance. First, we tested metric (or weak) invariance by comparing the unconstrained model with a model that constrains factor loadings to be equal across groups. Second, we tested scalar (or strong) invariance by comparing the metric model to one that additionally constrains item intercepts to be equal. Finally, we tested strict invariance by comparing the scalar model to a model that also constrains residuals to be equal across tested groups. As recommended by Cheung and Rensvold (2002), we evaluated model comparisons using the χ^2^-test as well as differences in *CFI* and gamma hat (*GH*; Steiger, 1989). χ^2^ should ideally not be significant, and Δ*CFI* and Δ*GH* should not be larger than 0.01 between models. As per recommendations from Trizano-Hermosilla and Alvarado (2016), we reported McDonald's ω (McDonald, 1999) as a measure of internal consistency. To compare the SREBQ by sociodemographic groups, we computed ANOVAs.

## Results

### Item and Scale Characteristics

We ran this descriptive analysis in Sample 1. The Shapiro-Wilk test for normal distribution was highly significant, *p* < 0.001, for all items and the scale. However, considering our sample size, this was not surprising (Yap and Sim, 2011; Kim, 2013). For larger samples (*n* > 300), Kim recommends the analysis of skewness and kurtosis by comparing empirical values to the cutoff values of two for skewness and four for excess kurtosis. Thus, the data can be considered normally distributed. Reliability for the 5-Item scale was ω = 0.652. We reported further descriptive statistics in Table 2.

### Factor Structure

In the initial PA, the empirical eigenvalues for the first three components were 2.02, 0.89, and 0.80, whereas the randomly generated ones were 1.21, 1.11, and 1.03. Thus, it was confirmed that only a singular component should be extracted from the five-item matrix. We reported the factor loadings of the subsequent EFA in Table 2. Upon inspection of the various item descriptive statistics, it became evident that Item 5 is not a very good measure of the underlying construct. Its factor loading in EFA and its corrected item-total correlation are below 0.30.

Next, we conducted CFA (see Table 3). In Sample 1, the model proposed by Kliemann et al. (2016) had moderate to unacceptable fit, which led us to seek for a better fitting model. We found high modification indices for Item 5, indicating non-factor specific correlations with other indicators. This combined with the low CFA factor loading (λ = 0.165), low EFA loading, and the low-item-total correlation result in a marked improvement when Item 5 was excluded from the scale. ω for the 4-Item scale is 0.735. The remaining CFA factor loadings were then between 0.365 and 0.955.

**Table 3:** Confirmatory factor analysis results of the Self-Regulation of Eating Behavior Questionnaire (Samples 1 and 2).

**Sample**	**Model**	**χ^2^(*df*)**	***p***	**CFI**	**TLI**	**RMSEA [90% CI]**	**SRMR**
1	Original model	21.63 (5)	0.001	0.956	0.913	0.097 [0.058; 0.141]	0.056
	Adapted model	1.51 (2)	0.471	1.000	1.004	0.000 [0.000; 0.096]	0.013
2	Original model	74.413 (5)	< 0.001	0.976	0.951	0.079 [0.064; 0.096]	0.039
	Adapted model	5.96 (2)	0.051	0.999	0.996	0.030 [0.000; 0.059]	0.009
Simulated cutoffs	Original model	11.28		0.998	0.996	0.022	0.013
	Adapted model	6.46		0.999	0.996	0.030	0.011

*χ^2^ is Yuan-Bentler-scaled. Original model is the one proposed by Kliemann et al. (2016). Adapted Model excludes Item 5*.

This initial analysis was replicated in Sample 2. Here we found good model fit for both, the 5- item and the 4-item version of the SREBQ. Internal consistency was acceptable for both as well (ω_5_ = 0.700; ω_4_ = 0.766). It should be noted that Item 5 again displayed a very low factor loading, λ = 0.065. Thus, it cannot be recommended for the assessment of self-regulation of eating behavior within the German SREBQ. Consequently, we excluded item 5 from the model. The other loadings fell between 0.334 and 0.882. We supplemented these analyses by comparing the empirical model fit indices to simulated cutoff values generated in the R package ezCutoffs (Schmalbach et al., 2019). As is evident from Table 3, the adapted model is within the acceptable bounds laid out by the 95th percentile of the simulated fit index distribution, but the original model is not.

Next, we only tested for measurement invariance in Sample 2. We found evidence for strict measurement invariance across participant sex as well as age and BMI (Kg/m^2^) groups, as evidenced by small differences in CFI and GH (see Table 4). The χ^2^-test was significant in four of the nine comparisons, however it is well-known to be overly sensitive in larger sample sizes (Bentler and Bonett, 1980). The difference in *CFI* and *GH* did not exceed 0.01 for any of the comparisons. Only the test of strict invariance in the case of the weight status groups was not fully conclusive with significant Δχ^2^ and ΔCFI but an insignificant ΔGH. For this variable, only scalar invariance should be assumed. In sum, the measurement models were fully equivalent across age and sex, and largely equivalent for weight status.

**Table 4:** Analysis of measurement invariance (Sample 2).

**Model**	**χ^2^(*df*)**	**Δχ^2^**	**Δ*df***	***p***	***CFI***	**Δ*CFI***	***GH***	**Δ*GH***
**Gender mutligroup analysis (female/male)**								
Configural invariance	7.11 (4)				0.999		0.999	
Metric invariance	18.35 (7)	11.24	3	0.010	0.996	0.003	0.998	0.001
Scalar invariance	37.95 (10)	19.60	3	<0.001	0.990	0.006	0.994	0.004
Strict invariance	43.49 (14)	5.54	4	0.236	0.988	0.002	0.994	0.000
**Age multigroup analysis (14**–**29, 30**–**39, 40**–**49, 50**–**59, 60**–**69**, **≥70)**								
Configural invariance	13.66 (12)				0.999		10.000	
Metric invariance	33.73 (27)	200.07	15	0.169	0.998	0.001	0.999	0.001
Scalar invariance	57.17 (42)	23.44	15	0.075	0.995	0.003	0.997	0.002
Strict invariance	97.54 (62)	40.37	20	0.004	0.986	0.009	0.993	0.004
**Weight status multigroup analysis (<25, 25**–**30**, **>30)**								
Configural invariance	8.768 (6)				0.999		0.999	
Metric invariance	9.733 (12)	0.965	6	0.987	1.000	0.001	1.000	0.001
Scalar invariance	21.174 (18)	11.441	6	0.076	0.999	0.001	0.999	0.001
Strict invariance	59.671 (26)	38.497	8	<0.001	0.985	0.014	0.993	0.006

### Convergent and Divergent Validity

We calculated correlations between the SREBQ and the other instruments for which we collected data in Sample 1. All associations were significant (*p* < 0.05), most highly so (*p* < 0.001; see Table 5). The SREBQ exhibited moderate negative correlations with the EDE-Q8, the SCL-9-K, and both subscales of the PHQ-4. It correlated weakly and positively with SWLS and the Health VAS. Finally, we found moderate to high negative associations with the subscales of the 6FQ. In addition, we calculated a correlation between BMI (kg/m^2^) and SREBQ in Sample 2. Here we found a moderately sized, negative correlation, *r* = −0.274, *p* < 0.001, indicating that individuals with higher BMIs have worse self-regulation of eating behavior.

**Table 5:** Correlations between the Self-Regulation of Eating Behavior Questionnaire and theoretically related scales (Sample 1).

	**1**	**2**	**3**	**4**	**5**	**6**	**7**	**8**	**9**	**10**	**11**	**12**	**13**
1—SREBQ	1.000	−0.187^***^	−0.296^***^	−0.207^***^	−0.215^***^	0.133^*^	0.156^***^	−0.469^***^	−0.279^***^	−0.513^***^	−0.257^***^	−0.249^***^	−0.140^**^
2— EDE-Q8		1.000	0.418^***^	0.295^***^	0.292^***^	−0.262^***^	−0.290^***^	0.008	0.298^***^	0.465^***^	0.051	0.724^***^	0.537^***^
3—SCL-9-K			1.000	0.669^***^	0.759^***^	−0.426^***^	−0.435^***^	0.331^***^	0.472^***^	0.310^***^	0.154^**^	0.544^***^	0.242^***^
4—PHQ-4 Depression				1.000	0.706^***^	−0.527^***^	−0.425^***^	0.233^***^	0.403^***^	0.253^***^	0.194^***^	0.433^***^	0.183^***^
5—PHQ-4 Anxiety					1.000	−0.425^***^	−0.402^***^	0.238^***^	0.407^***^	0.264^***^	0.098	0.409^***^	0.149^**^
6—SWLS						1.000	0.470^***^	−0.193^***^	−0.341^***^	−0.136^*^	−0.169^**^	−0.369^***^	−0.139^*^
7—Health VAS							1.000	−0.180^**^	−0.251^***^	−0.126^*^	−0.240^***^	−0.357^***^	−0.163^**^
8—6FQ Convenient Diner								1.000	0.441^***^	0.259^***^	0.252^***^	0.185^***^	0.082
9—6FQ Fast Pacer									1.000	0.335^***^	0.213^***^	0.385^***^	0.316^***^
10—6FQ Easily Enticed Eater										1.000	0.138^*^	0.423^***^	0.325^***^
11—6FQ Exercise Struggler											1.000	0.193^***^	0.049
12—6FQ Self-Critic												1.000	0.488^***^
13—6FQ All-Or-Nothing Doer													1.000

*** = significant at p < 0.001,

** = significant at p < 0.01,

* * = significant at p < 0.05*.

### Diagnostic Validity and Sociodemographic Variables

We compared sociodemographic groups by calculating ANOVAs in Sample 2. All effect sizes (η^2^_*p*_) indicated small to less than small effects, as per Cohen (1992). Only weight status was substantially associated with SREBQ, with medium effect size (see Table 6). Participants with a BMI between 25 and 30 kg/m^2^ indicative of overweight exhibited lower levels of self-regulation than individuals with a BMI lower than 25 kg/m^2^, displaying normal weight or underweight, *t*(2,151) = 9.15, *p* = 0.001. Also, participants with a BMI higher than 30 kg/m^2^ in the obese weight range scored lower on the SREBQ scale than participants with a BMI lower than 25 kg/m^2^, *t*(1,425) = 12.65, *p* = 0.001. Further, the results indicated that women scored significantly higher in the SREBQ than men. In addition, individuals with a higher degree of education and income exhibited higher levels of self-regulation of eating behavior. Derived norm values are provided in Table 7.

**Table 6:** ANOVA results for the Self-Regulation of Eating Behavior Questionnaire (Sample 2).

	***F***	***p***	**η^2^***_***p***_*
Gender	20.54	<0.001	0.008
Age	2.07	0.067	0.004
Education	4.51	0.001	0.007
Family	2.28	0.044	0.004
Employment	1.27	0.281	0.002
Income	3.80	0.043	0.006
Weight status	91.18	<0.001	0.074

**Table 7:** Normative percentile ranks for the Self-Regulation of Eating Behavior Questionnaire sum scores.

	**Male**			**Female**		
**Weight status**	**<25**	**25–30**	**>30**	**<25**	**25–30**	**>30**
**Sum score**	***n*** **= 476**	***n*** **= 601**	***n*** **= 156**	***n*** **= 621**	***n*** **= 455**	***n*** **= 174**
4	0	0	1	0	0	2
5	0	1	1	0	2	3
6	1	2	2	0	3	6
7	3	5	5	1	5	9
8	7	10	17	3	9	18
9	11	15	31	6	15	27
10	18	23	42	10	22	36
11	26	35	57	17	35	49
12	40	49	67	30	47	59
13	53	64	77	44	59	71
14	65	78	88	55	73	84
15	75	86	93	67	80	88
16	84	93	97	80	89	95
17	92	96	99	91	96	98
18	94	98	99	95	98	98
19	98	99	100	97	99	100
20	100	100	100	100	100	100

## Discussion

The aim of the present study was to develop a German version of the SREBQ and to assess the unifactorial model of the scale to German data. For this aim, we first tested the five translated items in a model corresponding to the one proposed by Kliemann et al. (2016).

Our data exhibited medium to poor fit in the original model across the employed indices (except for SRMR). Additionally, Item 5 had a very low item-total correlation and factor loading, leading us to exclude it and test an adapted model consisting of the remaining four items. This is consistent with the results of the original model, since item 5 also showed a smaller loading relative to the remaining items (Kliemann et al., 2016). Still, a possible explanation of this result might lie in the German translation and wording of this item 5 (“If I am not eating in the way I intend to I make changes”). It is conceivable that the word “intention” and the notion of it might have led to different responses. For example, to have an (initial) plan or an intention is not the same (Ajzen, 1985; Gollwitzer and Sheeran, 2006) opening scope for divergent interpretations of these concepts. A more precise translation, such as “… the way I *initially planned* to” might have provided a more accurate response in the German population. An alternative perspective provides cross-cultural differences related to food choices and health beliefs (e.g., quality vs. quantity; Leeman et al., 2011; Meule, 2020). In contrast to the fice-item model, the four-item model evidenced exceptional fit indices across the board and was thus accepted. Item characteristics for Items 1–4 were adequate and scale reliability improved to ω = 0.738, which is an acceptable internal consistency.

In a following study (Study 2), we replicated these findings in a robust and representative sample of the German population (Sample 2). In addition, we employed a simulation-based algorithm for judging model fit (Schmalbach et al., 2019). This approach addresses criticisms of the traditional fixed cutoff values (Nye and Drasgow, 2011) and hence presented conclusive support for the model fit of the 4-item SREBQ over the 5-item version. Furthermore, we presented evidence for strict invariance of the SREBQ across participant sex and age. Concerning weight status, we found evidence of scalar invariance, which is the minimum prerequisite for meaningful group comparisons.

The correlations of the SREBQ with related measures demonstrated convergent validity. First and foremost, we found moderate to high negative associations with the 6FQ, which captures unhealthy nutritional lifestyle choices—the opposite of successful self-regulation of eating behaviors. Furthermore, we observed negative relationships with eating disorder psychopathology, as well as psychological distress and more general psychopathology—depression and anxiety, confirming the reports of Tangney et al. (2004). On the other hand, positive correlations with life satisfaction and subjective health status were exhibited, as reported in previous studies (e.g., Gupta and Verma, 2019; Sharbafshaaer, 2019). Finally, there were no meaningful differences in SREBQ with regard to sociodemographic variables, except for gender. This indicates that other variables, such as personal lifestyle choices (as evidenced by the 6FQ) might be of greater interest in explaining interindividual differences in SREBQ. In line with previous studies (Campos-Uscanga et al., 2017; Johnson and Annesi, 2018), our results suggested that individuals with overweight and obesity exhibited lower levels of self-regulation than individuals with normal weight or underweight. Further, women exhibited better self-regulation of eating behavior than men. This outcome is in accordance with previous evidence, reporting that compared to men, women had better self-regulation in terms of nutritional behavior (Anderson et al., 2007). A possible explanation could be that women feel more social pressure concerning their appearance (Helfert and Warschburger, 2013), are in average more concerned with dieting and have a higher health literacy than men (Rozin et al., 2003). Moreover, our results suggested that individuals with a higher degree of education and income have in average higher levels of self-regulation of eating behavior. This outcome is consistent with the construct of health-related locus of control (Strudler Wallston and Wallston, 1978), which is related to self-regulation (Bandura, 1977; AbuSabha and Achterberg, 1997; Scoffier et al., 2010). It describes the ability to control one's health is a relevant determinants of health behavior and it has shown to be predictive of nutrition, weight control, and compliance to health programs, as described in past studies (Steptoe and Wardle, 2001; Helmer et al., 2012; Anastasiou et al., 2015).

### Limitations

A potential criticism of our study is that we conducted both EFA and CFA on Sample 1. It is generally recommended to avoid doing so in order to avoid model over-specification. Nonetheless, this criticism can be invalidated by the confirmatory analysis we conducted in Sample 2. From the beginning, Sample 1 was planned as an exploratory pilot study, whereas the much larger Sample 2 was collected for purely confirmatory purposes. Further, even if the four-item-model yielded an adequate fit, it needs to be noted that item 4 exhibited a comparatively small loading. Future research could also consider testing the original scale with an adapted wording of the item 5. Taking these aspects into account further studies are needed to confirm our results.

Even if we did find a general negative association between self-regulation of eating behavior and eating disorder pathology (and psychopathology in general), we did not differentiate between types of eating disorders. EDs are associated with a disturbed self-regulation of eating behavior. While anorexia nervosa is related to an excessive and thus pathological amount of self-regulation, bulimia nervosa and binge-eating disorder (Hagger et al., 2009; Dingemans et al., 2017; Kenny et al., 2017, 2019) were found to be associated with lower levels of self-regulation. Consequently, we assumed that the SREBQ would evince these specific differences in the self-regulation of eating behavior. The negative association between SREBQ and BMI (kg/m^2^) is indicative of that, confirming this assumption. On a related note, the immediate impact of self-regulation on BMI trajectory would be an interesting research topic.

## Conclusion

The data collected for the SREBQ assessment revealed that is a suitable tool for the measurement of an adult's and adolescent's self-regulatory ability in terms of eating behavior. In addition, a refined model was more suitable. With four, rather than five items the SREBQ allows for an economical yet accurate evaluation and can be recommended for medical, psychological, and nutritional research.

## Data Availability Statement

The datasets generated and/or analyzed in Study 2 during the current study are not publicly available due to data protection, but are available from the authors who sponsored the data collection (Prof. Brähler, Prof. Hilbert, Prof. Beutel) upon reasonable request.

## Ethics Statement

Ethics approval and consent to participate Study 1: The ethics committee of the University of Applied Sciences Magdeburg-Stendal approved of the procedure (AZ-4973-75). We conducted the study in accordance with the common ethical guidelines of the Declaration of Helsinki. Ethics approval and consent to participate Study 2: The ethics committee of the University of Leipzig approved the procedure (002/20-ek). Both studies were conducted in accordance with the common ethical guidelines of the Declaration of Helsinki.

## Author Contributions

EB and AH contributed with data collection and study design and provide advice. MZ and KP contributed with literature research and provided critical advice for data interpretation. IS and BS contributed with writing the first draft of the paper. BS contributed with data analysis and interpretation and provided helpful advice for the discussion. All authors contributed with the writing and drafting of the paper.

## Conflict of Interest

The authors declare that the research was conducted in the absence of any commercial or financial relationships that could be construed as a potential conflict of interest.
